# Sugar-sweetened beverage consumption among pregnant women attending general and teaching hospitals in Ibadan, Nigeria: SSB consumption during pregnancy

**DOI:** 10.1186/s12889-023-15828-z

**Published:** 2023-05-26

**Authors:** Ikeola Adeoye

**Affiliations:** 1 Department of Epidemiology and Medical Statistics, Faculty of Public Health, College of Medicine, University of Ibadan, Ibadan, Nigeria; 2 Consortium for Advanced Research Training in Africa (CARTA), Nairobi, Kenya

**Keywords:** Sugar sweetened beverages, Pregnancy, Frequency, Factors, Ibadan

## Abstract

**Background:**

Sugar-sweetened beverages (SSBs) have become a global health concern because of their adverse health effects and their association with the obesity pandemic. It has not received much attention in sub-Saharan Africa, including Nigeria, especially among pregnant women. The pattern, frequency and factors associated with SSBs among pregnant women in Ibadan, Nigeria, were investigated.

**Design:**

Data were from the Ibadan Pregnancy Cohort Study - a prospective cohort study investigating 1745 pregnant women from four comprehensive obstetric facilities in Ibadan. A qualitative food frequency questionnaire (FFQ) was used to assess the pregnant women’s intake of food and drinks over the previous months. Sugar-sweetened beverage variable and scores were also generated using the principal component analysis with varimax rotation. Factors associated with high SSB scores were examined using multivariate logistics regression analyses at a 5% significance level.

**Results:**

The most commonly consumed SSBs were cocoa-sweetened beverages, soft drinks, malt drinks, and fruit juice. A quarter of the women (75th percentile) consumed SSB more than once weekly. The factors associated with high SSB on multivariate analysis were; being employed (AOR: 1.52, 95% CI 1.02–2.26), maternal obesity (AOR: 0.065, 95% CI 0.47–0.89), high fruit intake (AOR:3.62, 95% CI 2.62–4.99), high green vegetable consumption (AOR:1.99, 95% CI 1.06–3.74), high milk intake (AOR: 2.13, 95% CI 1.65– 2.74), frequent fast food outlet visit (AOR: 2.19, 95% CI 1.53–1.70), all of these remained significant after adjusting for confounding variables.

**Conclusion:**

SSBs were common among our study population. Factors associated with high SSBs intake are crucial for implementing locally relevant public health interventions.

**Supplementary Information:**

The online version contains supplementary material available at 10.1186/s12889-023-15828-z.

## Introduction

Sugar was absent in the human diet until about the 14th century but has become a dominant component of foods and drinks in the last three centuries, first in Western countries and now consumed globally as added sugars and SSB [1–4]. SSB consumption has become a significant public health issue, particularly in low and middle-income countries (LMIC) undergoing rapid urbanisation, economic growth, marketing and nutrition transition [5–7]. SSBs are nonalcoholic beverages with various added sugars, including brown sugar, corn sweetener, corn syrup, dextrose, fructose, glucose, high-fructose corn syrup, honey and so on [8, 9]. They include carbonated soft drinks, fruit juices, sweetened water, sports drinks, energy drinks, coffee and tea with added sugars. Naturally occurring sugars such as fruits and milk are not considered SSBs [10]. SSBs have been strongly linked to the obesity pandemic and other adverse health effects [1, 6, 11, 12, 13]. They are also independently associated with weight gain, type 2 diabetes mellitus, dyslipidemia, cardiovascular diseases, nonalcoholic fatty disease, gout and an increased risk of premature death in adults [14–16]. SSBs have a high content of readily absorbable sugar associated with low satiety and poor compensation for total energy, contributing to excess energy intake stored up as body fat [1, 17, 18]. Fortunately, SSB consumption is a modifiable risk factor for obesity and the associated co-morbidities, and can be targeted for public policy and interventions.

The potential biological mechanisms by which habitual SSBs results in weight gain and other adverse health outcomes include low satiety after intake compared with solid food, consumption of large volume, and a poor compensatory reduction in energy intake at the next meal following the intake of liquid calories leads to a greater energy intake and weight gain [17]. SSBs also have addictive-like behaviours similar to cocaine addiction, characterised by intake, withdrawal and cravings, which result in sugar addiction and excessive consumption [19, 20]. Additionally, SSBs adversely affect metabolism by causing sudden elevation of blood glucose and insulin levels (spikes), particularly when taken in large volumes, resulting in high glycemic load leading to decreased insulin sensitivity, insulin resistance, inflammation, and beta-cell dysfunction [21, 22] which increases the risk of T2DM [4, 23, 24] and CVD [25, 26]. Additionally, the fructose component has metabolic effects on the liver, which increase visceral adiposity, dyslipidemia and non-fatty liver disease because the hepatic metabolism of fructose favours denovo lipogenesis [1, 24, 27].

The WHO recommends limiting free sugars, including added sugars, to less than 10% of daily intake [28]. Pregnant women reportedly consume more sugar than their non-pregnant counterparts, partly because of increased energy demands and pregnancy-induced food cravings [29]. SSB consumption during pregnancy has been associated with poor diet [30] and greater total energy intake [26, 30, 31], excessive gestational weight gain [32–34], a higher risk of preeclampsia, gestational diabetes mellitus and gestational hypertension [25, 29, 30, 35] preterm delivery [36, 37] obesity in offspring and greater weight-for-age at birth [30, 36]. SSB during pregnancy is thought to have an intrauterine programming effect on the fetus, which increases the risk of developing noncommunicable diseases in childhood, including childhood obesity [38–41], childhood asthma [42] and reduced cognitive ability [43].

The reported consumption of sugar-sweetened beverages among pregnant women ranges from 21.9 to 81% in developed countries [10, 29]. Studies have reported the factors associated with maternal SSB consumption, including pre-gravid tobacco use, being unmarried, low income, maternal age, parity, and socioeconomic status [10, 37]. Other risk factors for SSB consumption during pregnancy include younger maternal age, lower education, household income, parity, socioeconomic status, country of birth and prolonged television viewing [30, 39, 44]. However, this evidence emanated from developed countries, mainly North America and Europe. Maternal consumption of SSBs has not received much attention in sub-Saharan Africa, including Nigeria.

**Fig. 1:**
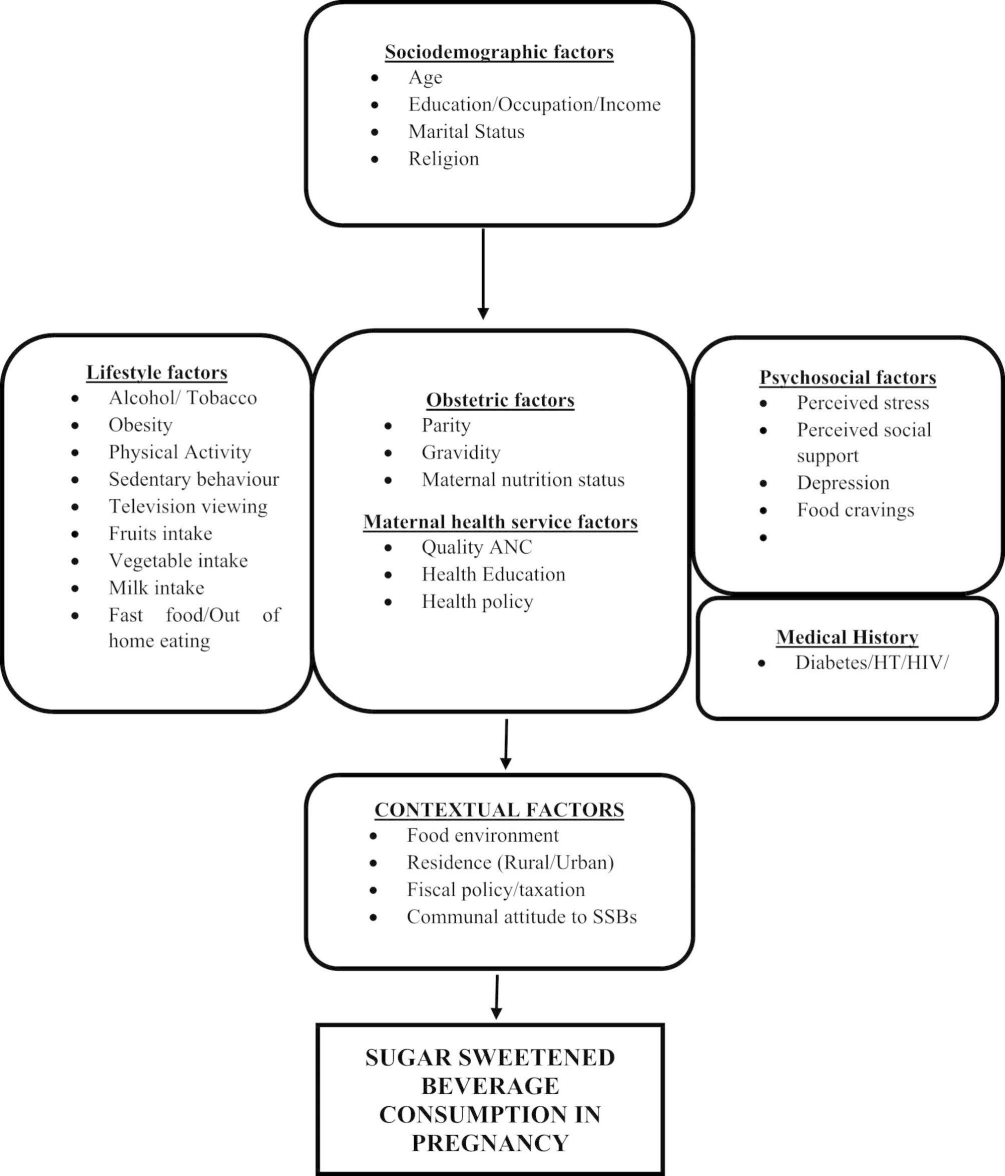
Conceptual framework for sugar-sweetened beverage consumption in pregnancy

Nigeria is undergoing a nutritional transition characterised by a change in the food consumption pattern from the healthy, traditional unrefined diet rich in fibre, tubers, fruits and vegetables to more processed foods high in added sugars, salt and saturated fat [45, 46]. Nigeria and other LMICs are the targets of the processed food industry due to weaker regulations and a more favourable policy environment for their profit-generating motives. Hence, the rapid spread of fast food restaurants and out-of-home eating in Nigeria contributed to changing food consumption patterns [45, 47], including SSB consumption among pregnant women, which has both short- and long-term intergenerational consequences. Studies on SSB consumption, especially among pregnant women, are lacking in Nigeria. Thus, this study investigated the pattern, frequency and factors associated with SSB consumption among pregnant women in Nigeria using a conceptual framework (Fig. 1). The framework facilitated the identification of associated critical individual and contextual level factors to enhance the understanding of SSB consumption among Nigerian women.

## Materials and methods

### Study design, setting and population

This study was a part of the Ibadan Pregnancy Cohort Study (IbPCS). The IbPCS was a prospective cohort study investigated the influence of maternal obesity and lifestyle characteristics on maternal glycaemia, gestational weight gain, and pregnancy outcomes in Ibadan, Nigeria. We implemented the survey from April 2018 to September 2019. The participants, methods, study design, sample size estimation and measures have been previously described [48]. In brief, the IbPCS is a prospective cohort study conducted among 1745 pregnant women that were recruited in early gestation (gestational age ≤ 20 weeks) from four comprehensive obstetric health facilities that provide referral care within the Ibadan metropolis: University College Hospital, Adeoyo Maternity Teaching Hospital, Jericho Specialist Hospital, Saint Mary Catholic Hospital, Oluyoro, Ibadan. Participant characteristics, namely, sociodemographic, household assets, and past obstetric and medical histories, were also documented at enrollment. Also, lifestyle characteristics were assessed, including dietary habits, sugar-sweetened beverage consumption, physical activity, sleep pattern, tobacco exposure, alcohol consumption, and women’s anthropometric measures (BMI). Study participants were followed up from enrollment through the second and third trimesters up to delivery. In addition, biomarkers, i.e. blood glucose and lipids, were assessed at “24–28 weeks”, while utilisation of maternal health care services, health care behaviour, serial maternal weight and pregnancy complications were evaluated in the third trimester. The individual-level factors associated with SSB consumption among pregnant women were examined using the conceptual framework in Fig. 1.

### Data collection

#### Beverage intake assessment

A qualitative food frequency questionnaire (FFQ) was used to assess the pregnant women’s intake of food and drinks over the previous months. The FFQ was made of 67 food and beverages classified into food groups, including SSB consumption. The development and validation of the FFQ and dietary pattern among pregnant women have been described elsewhere [49].

The beverage assessment was a one-time cross-sectional assessment among the study participant. The beverages included creamed milk, low-fat milk, soya milk, “kunu”, soft drinks, malt drinks, fruit juice, milo, tea, coffee, yoghurt, beer, palm wine, and whisky/dry gin. The frequency of consumption of each beverage was grouped into the following categories: once daily, more than once daily, once a week, more than (> 1) once a week, once in a month, more than once a month (> 1), rarely/never. The sugar-sweetened beverage variable was created using the frequency of consumption of cocoa-sweetened beverages, soft drinks, malt drinks and fruit juices, which were the most commonly consumed SSBs in our study population. The “sugar-sweetened beverage” variable was grouped into four categories (heavy - “1 once daily or 2–3 times daily”; frequently - “1 once weekly or 2–3 times weekly”; occasionally - “1 once monthly or 2–3 times monthly”; rarely - “rarely or never”. Principal component analysis with varimax rotation was used to generate sugar-sweetened beverage scores. The SSB scores were grouped into tertiles and median for further analysis. High SSB intake was defined as SSB scores ≥ the 50th percentile and low if less than the 50th percentile. The other diet-related co-variates examined in this study were fruits, milk, vegetable intake and eating from fast-food outlets. Fruits examined included pawpaw, watermelon, pineapples, apples, tangerine, cucumber, avocado, pear, oranges, carrots, mangoes, banana and cherry. Milk consisted of cream milk, skimmed milk and soya milk. Fruit and milk scores were derived using the principal component analysis with varimax rotation categorised into tertiles and median. The frequency of vegetable intake and eating at fast food outlets were captured as often (weekly or more) or seldom (monthly or less).

#### Variable measurement

A structured, pretested interviewer-administered questionnaire was used to collect information on sociodemographic and lifestyle characteristics, including physical activity, anthropometric measurement (weight in kg, height in metres), and Physical activity (METs). The physical activity level was assessed using the Pregnancy Physical Activity Questionnaire (PPAQ) [50]. Total activity in metabolic equivalents (METs) and the duration of moderate-intensity exercise in minutes were estimated using the PPAQ guidelines [50]. Maternal BMI (kg/m2) was a function of weight (kg) divided by height squared (m2) and was defined based on the WHO classification: underweight (≤ 18.5 kg/m2), normal weight (18.5–24.9 kg/m2), overweight (25.0- 29.9 kg/m2), obesity (≥ 30 kg/m2) [51, 52]. Perceived stress was measured using the Perceived Stress Scale (PSS) [53]. The individual’s perceived stress (maximum score of 40) was categorised into low stress (0–13), moderate stress (14–26) and high stress (27–40).

### Data processing and analysis

Data were analysed using STATA version 13. The primary dependent variable was SSB intake. Numeric variables were summarised with means and standard deviation, and categorical variables were summarised with proportions and percentages. Composite bar graphs were used to present the frequency and types of SSB consumption among pregnant women, which is the outcome variable in this study. The explanatory variables included maternal age, level of education, religion, marital status, monthly income, marital status, gravidity, parity, tobacco exposure, alcohol consumption, physical activity, time spent in television viewing, fruits, vegetable and milk intake, fast food outlet, perceived stress, chronic illness and the number of ANC visits. The association between maternal characteristics and the frequency of SSB intake was assessed using chi-square for categorical variables and ANOVA for continuous variables at a 5% level of statistical significance. Binary logistic regression analyses were conducted to identify the factors associated with high intake of SSB among pregnant women Factors that were statistically significant at a 5% binary logistic analysis were used to fit the multivariate analysis. A Forest plot was employed to help visualise the adjusted odds ratios of factors associated with high SSB intake derived from the multiple logistic regression analysis.

## Results

### The characteristics of study participants by their SSB consumption

The characteristics of the pregnant women by their degree of SSB consumption are shown in Table 1. The mean age at enrolment and early pregnancy BMI were 29.8 years and 25.7 kg/m2, respectively. The SSB consumption decreased significantly with maternal age (p = 0.026) and maternal BMI (p < 0.001) but had a positive association with total physical activity level (p = 0.012) and the duration of moderate-intensity activity (p < 0.001). Notably, younger women under 35 (7.85%) had a significantly higher SSB intake than older women above 35 (6.46%). Heavy consumption of SSB also declined in a dose-response fashion by maternal BMI: underweight (14.0%), normal weight (8.3%), overweight (6.6%), and obese (5.2%).

### Frequency of SSB consumption among pregnant women in Ibadan

**Fig. 2:**
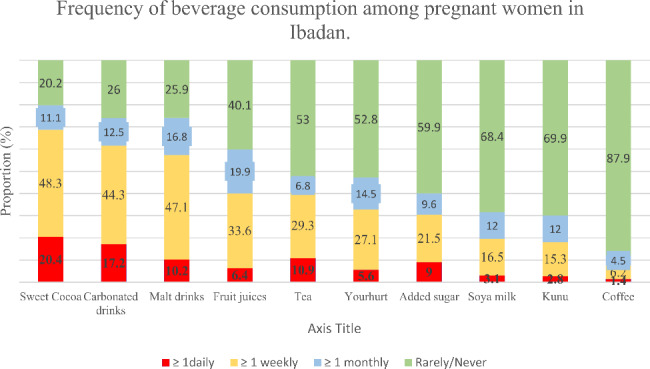
Frequency of consumption of sugar-sweetened beverages among pregnant women in Ibadan

The types and patterns of SSB consumption among pregnant women in Ibadan are shown in Fig. 2. The most commonly consumed SS were sweetened chocolate drinks (20.4% daily and 48.3% weekly), soft drinks (17.2% daily ad 44.3% weekly), malt drinks (10.2% daily and 47.1% weekly) and fruit juices (6.4% daily and 33.6% weekly). Consumption of coffee was negligible among the study participants (1.4% daily and 6.2% weekly). About 50% and 25% of the women consumed SSB more than once monthly and more than once weekly, respectively.

### Factors associated with high intake of SSB

**Fig. 3:**
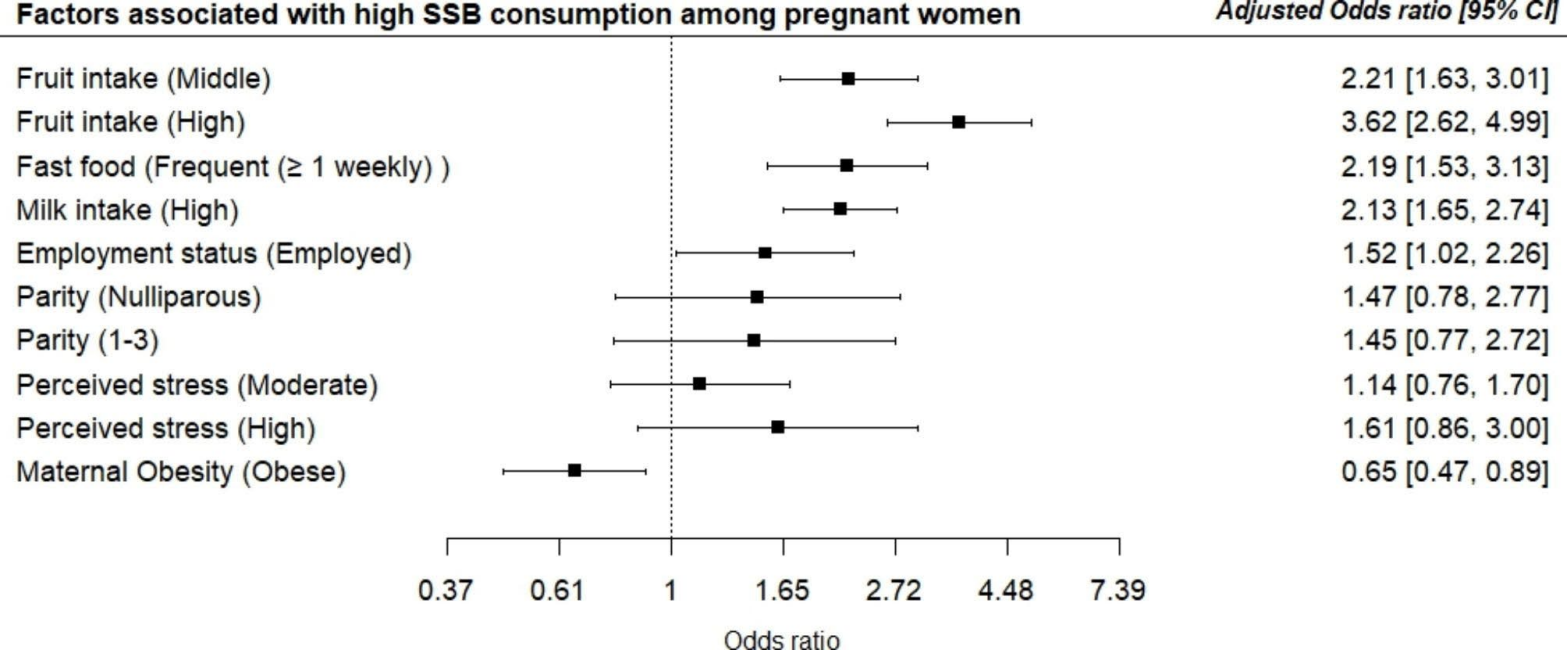
Forest plot showing the factors associated with high SSB consumption among pregnant women in Ibadan, Nigeria

The factors associated with a high intake of SSB are represented in Table 2. On binary logistic analysis being employed, parity, maternal obesity, fruits, vegetables and milk intake, eating from fast food outlets and perceived stress were associated with high SSB consumption. Specifically, women that were employed (OR: 1.46, 95% CI 1.07–1.99), nulliparous (OR: 1.59, 95% CI 1.02–2.48), high level of stress (OR: 2.02, 95% CI 1.17–3.52) had a high intake of SSB. Conversely, women with obesity had a low likelihood of high SSB intake (OR: 0.66, 95% CI 0.52–0.85). High fruit, vegetable, and milk intake were also associated with high SSB consumption. On multivariate analysis being employed (AOR: 1.52, 95% CI 1.02–2.26), high fruit intake (AOR: 3.62, 95% CI 2.62–4.99), high green vegetable consumption (AOR: 1.99, 95% CI 1.06–3.74), high milk intake (AOR: 2.13, 95% CI 1.65–2.74), frequent fast food outlet visit (AOR: 2.19, 95% CI 1.53–1.70). Maternal obesity remained a significant protective factor for high SSB intake (AOR: 0.065, 95% CI 0.47–0.89) after adjusting for confounding variables. Forest plots displaying the adjusted odds ratios and 95% confidence intervals of the factors associated with high SSB consumption during pregnancy are presented in Fig. 3.

**Table 1:** Characteristics of pregnant women by the frequency of sugar-sweetened beverages intake in Ibadan, Nigeria

Characteristics	Category of Maternal SSB intake									P-value
	Total	Rarely/Never		Occasionally		Frequently		Heavy		
**Mean (SD)**										
Age at enrolment (years)	29.8 (5.4)	30.8 (5.8)	30.2 (5.2)		29.7 (5.5)		29.0 (4.9)		*0.026	
Maternal BMI (kg/m^2^)	25.7 (5.3)	27.4 (5.6)	25.9 (5.3)		25.5 (5.1)		24.8 (5.7)		*0.001	
Total activity (METs)	290.5(124.9)	285.6 (144.8)	280.9 (123.4)		292.2 (119.8)		320.3(143.2)		0.012	
Moderate intensity activity (minutes)	26.3 (22.9)	27.1 (28.4)	23.6 (21.9)		26.4 (21.6)		34.6 (28.0)		*0.000	
** N 1745 (%)**										
**Age**										
Less than 35 years	1,389 (79.6)	82 (5.9)	421 (30.3)		777(55.9)		109 ( 7.9)			
35 and above	356 (20.4)	37 (10.4)	105 (29.5)		191 (53.7)		23 (6.5)		*0.024	
**Education**										
Primary or less	49 (2.8)	3 (6.1)	11 (22.5)		31 (63.3)		4 (8.2)			
Secondary	504 (29.0)	33 (6.6)	135 (26.8)		287 (56.9)		49 (9.7)			
Tertiary or higher	1188 (68.4)	83 (7.0)	379 (31.9)		648 (54.6)		78 (6.6)		0.132	
**Religion**										
Christianity	1010 (58.2)	68 (6.7)	302 (29.9)		572 (56.6)		68 (6.7)			
Islam	726 (41.8)	49 (6.8)	221 (30.4)		393 (54.1)		63 (8.7)		0.452	
**Marital status**										
Single	102 (5.8)	9 (8.8)	25 (24.5)		58 (56.9)		10 (9.8)			
Married	1643 (94.2)	110 (6.7)	501 (30.5)		910 (55.4)		122 (7.4)		0.469	
**Income (Naira)**										
< 20,000	583 (38.0)	30 (5.2)	166 (28.5)		331 (56.8)		56 (9.6)			
20,000–99,000	843 (55.0)	58 (6.9)	259 (30.7)		469 (55.6)		57 (6.8)			
100,000 and above	108 (7.1)	7 (6.5)	34 (31.5)		59 (54.6)		8 (7.4)		0.417	
**Employment status**										
Unemployed	189 (10.8)	20 (10.6)	64 (33.9)		94 (49.7)		11 (5.8)			
Employed	1556 (89.2)	99 (6.4)	462 (29.7)		874 (56.2)		121 (7.8)		0.058	
**Ethnicity**										
Non Yoruba	178 (10.2)	15 (8.4)	55 (30.9)		94 (52.8)		14 (7.9)			
Yoruba	1564 (89.2)	103 (6.6)	471 (30.1)		872 (55.8)		118 (7.5)		0.776	
**Parity**										
Nullipara	760 (43.8)	47 (6.2)	226 (29.7)		429 (56.5)		58 (7.6)			
Para ≥ 1	977 (56.3)	71 (7.3)	299 (30.6)		535 (54.8)		72 (7.4)		0.776	
**Gravidity**										
Primigravida	564 (32.5)	35 (6.2)	168 (29.8)		314 (55.7)		47 (8.3)			
2–4	983 (56.3)	64 (6.5)	299 (30.4)		552 (56.2)		68 (6.9)			
5 and above	191 (11.0)	19 (10.0)	57 (29.8)		99 (51.8)		16 (8.4)		0.550	
**BMI**										
Underweight	50 (3.0)	2 (4.0)	19 (38.0)		22 (44.0)		7 (14.0)			
Normal weight	845 (49.8)	44 (5.2)	236 (27.9)		495 (58.6)		70 (8.3)			
Overweight	473 (27.9)	34 (7.2)	150 (31.7)		258 (54.6)		31 (6.6)			
Obese	328 (19.3)	36 (11.0)	114 (34.8)		161 (49.1)		17 (5.2)		*0.001	

**Table 2:** Crude and adjusted odds ratios and 95% confidence intervals of factors associated with high SSB consumption among pregnant women in Ibadan, Nigeria

Characteristics	Crude OR (95% CI)	p-value	Adjusted OR (95% CI)	p-value
*Sociodemographic factors*				
**Age**				
Less than 35	1			
35 and above	1.03 (0.81–1.30)	0.818		
**Education**				
Primary or less	1			
Secondary	0.85 (0.46–1.57)	0.597		
Tertiary or higher	0.64 (0.79–2.56)	0.138		
**Employment status**				
Unemployed	1			
Employed	1.46 (1.07–1.99)	**0.017**	**1.52 (1.02–2.26)**	**0.041**
**Religion**				
Christianity	1			
Islam	1.04(0.85–1.26)	0.689		
**Marital status**				
Single	1			
Ever Married	1.02 (0.68–1.53)	0.937		
Less than 20,000	1			
20,000–99,999	0.82(0.66–1.01)	0.064		
100,000 and above	0.99 (0.65–1.50)	0.072		
**Parity**				
Nulliparous	1.59 (1.02 - 2.48)	**0.040**	1.47 (0.78–2.77)	**0.235**
1–3	1.52 (0.98–2.36)	0.063	1.45 (0.77–2.72)	0.246
4 and above	1		1	
*Lifestyle factors*				
**Maternal Obesity**				
Non-obese	1		1	
Obese	0.66 (0.52–0.85)	**0.001**	**0.65 (0.47–0.89)**	**0.008**
**Tobacco exposure**				
Yes	0.83 (0.50–1.36)	0.457		
No				
**Alcohol consumption**				
Yes	1.08 (0.81–1.44)	0.584		
No	1			
**Physical activity**				
Low				
High	1.07 (0.89–1.30)	0.466		
**Television viewing**				
< 3 h	1			
≥ 3 h	1.20 (0.99–1.45)	0.071		
**Fruits intake**				
low	1		**1**	
Middle	2.17 (1.70–2.76)	**< 0.001**	**2.21 (1.63–3.01)**	**< 0.0001**
High	5.26 (4.07–6.79)	**< 0.001**	**3.62 (2.62–4.99)**	**< 0.0001**
**Green vegetable intake**				
Low	1		1	
High	3.10 (1.99–4.81)	**< 0.001**	**1.99 (1.06–3.74)**	**0.028**
**Milk intake**				
Low	1		1	
High	3.18 (2.60–3.89)	**< 0.001**	**2.13 (1.65–2.74)**	**< 0.0001**
**Fast food**				
Seldom (≤ 1 month)	1			
Frequent (≥ 1weekly)	2.74 (2.06–3.64)	**< 0.001**	**2.19 (1.53–3.13)**	**< 0.0001**
*Psychological factors*				
**Perceived stress**				
Low	1		1	
Moderate	1.32 (0.93–1.88)	0.117	1.14 (0.76–1.70)	0.539
High	2.02 (1.17–3.52)	**0.012**	1.61 (0.86–3.00)	0.135
*Medical Condition*				
**Chronic Illness**				
Yes	0.73 (0.53–1.01)	0.054		
No	1			
*Maternal Health Service use*				
**ANC visits**				
< 4 visits	1			
≥ 4 visits	0.99 (0.73–1.36)	1.000		

## Discussion

Investigating SSB consumption among pregnant women in an LMIC country such as Nigeria fills an essential gap of public health significance because food and beverage consumption and maternal nutritional status have short- and long-term intergenerational consequences. Conversely, empirical evidence on SSB consumption, the factors associated and health outcomes have emanated mainly from Western countries, particularly among children, adolescents and adults [54, 55]. Hence, the frequency and factors associated with SSB consumption among pregnant women in Ibadan, Nigeria, were examined. SSB consumption was typical within our study population, with about a quarter of the pregnant women consuming SSB more than once weekly. This level of consumption is of concern because of the adverse health outcomes associated with SSB consumption which are poor diet quality, high total energy intake [30], excessive gestational weight gain [33, 34], hyperglycaemia and hyperinsulinemia [23, 24, 26]. Notably, the level of SSB consumption in our study is lower than those reported in developed countries [37, 56]. For example, Cheng et al. (2021) said 87.7% of pregnant women consumed SSBs more than once weekly among low-income women in the United States [56]. The high consumption of SSBs by pregnant women, particularly in our context, could be attributed to a lack of awareness of their harmful effects, assuming that, unlike alcoholic beverages, they do not threaten them or their babies. Sweetened chocolate drinks, carbonated soft drinks, malt drinks and fruit juices were our study population’s most commonly consumed SSB.

It was observed that maternal age and body mass index had an inverse relationship with the frequency of SSB consumption, while physical activity had a direct association. For example, younger women consumed SSBs more frequently than older women, and this inverse age gradient with SSBs is well reported in the literature [7, 30, 39, 57]. In contrast, pregnant women who engaged in physical activity tended to consume more SSBs to recover energy. Notably, the factors associated with SSB consumption during pregnancy across several domains were examined: sociodemographic, obstetric, lifestyle, maternal health service utilisation, and psychological factors. The significant factors associated with SSB consumption on bivariate logistic analysis were being employed, parity, maternal obesity, a healthy diet defined by a high intake of fruits, vegetables and milk intake, frequent fast food outlets and perceived stress. Socioeconomic factors have been reported to shape food choices [58]. In this study, employed women had a higher likelihood of high SSB consumption than women without employment. Employment ensures purchasing power; hence, employed women can afford SSBs. They are also more likely to engage in out-of-home eating, which increases the likelihood of large portion sizes, high palatability and high sugars, including SSB consumption [59]. In support of this finding, women who frequent fast-food restaurants were at least twice as likely to indulge in high SSB consumption during pregnancy as women who seldom patronise fast-food outlets. These associations remained after adjusting for confounding variables. Nulliparous women (AOR = 1.67) also consumed more SSBs than grand multiparous women. This may be because of inexperience, lack of awareness of the hazards of excess calorie intake during pregnancy and the myth of eating for two. Pregnancy-induced food cravings and other associated hormonal changes may increase women’s urge for SSB intake. Nulliparous women should be targets for health education and adopting a healthy lifestyle during antenatal care. Excess calorie intake during pregnancy leads to excessive gestational weight gain, post-partum weight retention, maternal obesity, and its attendant complications in subsequent pregnancies [60].

Importantly, we found an inverse relationship between SSB consumption and maternal obesity, whereas the direct association between SSB intake and obesity is well established in the literature [6, 12–14, 17, 18, 23, 24, 61]. Women with obesity were 35% less likely to have high SSB consumption (AOR = 0.65) than women who were not obese, even after adjusting for socioeconomic and lifestyle factors. Pregnant women are a particular group who are often motivated to adopt a healthy lifestyle to optimise their health and their baby. Hence, the inverse association could be reverse causation whereby obese women reduce their SSB intake to restrict weight gain and the associated complications. For instance, the United States Institute of Medicine GWG guideline stipulates the least allowable weight gain for obese women during pregnancy [62]. Besides, obese pregnant women may be on calorie restrictions or special diets by advice or recommendations. Other plausible reasons may include residual confounding from factors not measured or considered in the study and under-reporting of SSB intake by obese women because of a social desirability bias [63]. Temporality bias may not be ruled out because SSB consumption and maternal body mass index were measured at baseline. However, the inverse association between SSB and obesity have also been reported in the literature [64].

The association of specific dietary factors fruits, vegetables, milk, intake and eating at fast-food restaurants - with SSB consumption within the Nigerian context were examined. Surprisingly, high intakes of fruits, vegetables and milk (presumably healthy diets) were positively associated with high SSB consumption. This finding contradicts studies from Western countries that have reported a negative association between healthy dietary habits and SSB consumption among the population [64, 65]. This disparity may be due to a lack of awareness of the harmful effects of SSB among the populace. Food cravings during pregnancy may also drive the consumption of SSB among pregnant women.

Additionally, the Nigerian food environment in which SSBs are highly available, accessible and affordable, positive societal attitude towards SSB that considers it food, strong marketing and advertisement could influence the choice of SSBs among pregnant women. However, the association between fast food restaurants and SSB is similar to what has been reported by other studies [66, 67]. Hence, SSB consumption was associated with a healthy and unhealthy diet (fast foods). This finding attests to the pervasiveness of SSB consumption in which SSBs are regarded as food rather than a health threat to pregnant women indicating the importance of creating awareness of the hazards associated with SSB consumption among pregnant women to improve their dietary choices. The relationship between SSB and mental health issues recently gained attention among researchers. Our study found a positive association between stress and high SSB consumption, although the association became insignificant after adjusting for confounders.

Because SSBs are a modifiable risk factor, they should be targeted for policy and public health interventions among pregnant women [68, 69]. These interventions should include upstream policies such as the WHO best buy interventions, e.g. taxation, front-of-pack labelling, marketing restrictions on consumption patterns, and nutrition education campaigns to increase public awareness of the adverse effects of SSBs. The downstream approaches will include nutrition education at clinics to provide information on unhealthy diets, including SSBs, and replacing SSBs with alternative beverages, including water. Studies have shown that substituting water with SSBs leads to lower energy intake and weight loss [70, 71]. Sugar-sweetened beverage taxation has been recommended as an essential approach to related NCD prevention [72]. The WHO has proposed a 20% taxation on SSBs to reduce SSB consumption and raise revenue for disease prevention [73]. Some developed countries have successfully implemented SSB taxation. It has become a burning issue in sub-Saharan Africa because of the challenges (loss of revenue, unemployment and criminalising SSBs) associated with its implementation [74]. For these reasons, in a special issue, Ndlovu et al. (2021) explored the policy landscape for SSB taxation implementation in seven African countries [75]. Nigeria implemented the SSB taxation in 2022. However, local evidence on the health effects of SSB and the impact of SSB taxation is still lacking in several African countries [76].

This study is the first to investigate SSBs among the pregnant population in Nigeria, filling a critical research gap in Nigeria. This study could also inform public health policy in maternal health in Nigeria as it examined a broader range of factors compared with previous studies. However, both SSB intake and maternal BMI were assessed at baseline; hence temporality bias may not be ruled out. Also, the one-time cross-sectional assessment among the study participant did not allow the evaluation of the change in SSB consumption during pregnancy. Other limitations include self-reported measurement of SSB without estimating the portion size could be associated with misclassification bias. Future studies should examine the influence of SSBs on pregnancy outcomes.

## Conclusion

The pattern, frequency and predictors of SSBs among pregnant women attending general and teaching hospitals in Ibadan, Nigeria, were examined. The study participants commonly consumed SSBs. Maternal employment status, high fruits, vegetables and milk intake, and frequent fast food outlet visits were significant predictors of high SSB intake. This implies that SSB consumption is associated with a healthy and unhealthy diet (fast foods) in Nigeria because it is generally considered food, so it is frequently consumed. Conversely, maternal obesity was protective of high SSB intake due to caloric restriction by obese women, among other reasons. Stakeholders, especially nurses and physicians, should include SSB reduction in the nutrition education provided during antenatal care and other maternal nutrition awareness programmes.

## Electronic Supplementary Material

Below is the link to the electronic supplementary material.

Supplementary Material 1

## Data Availability

The datasets generated and/or analysed during the current study are not publicly available because they contain potentially identifying and confidential information but are available from the UI/UCH Ethics Committee (uiuchec@gmail.com) upon reasonable request if it meets the criteria for accessing confidential data.

## References

[1] Bray GA, Nielsen SJ, Popkin BM (2004). Consumption of high-fructose corn syrup in beverages may play a role in the epidemic of obesity. Am J Clin Nutr.

[2] Popkin BM, Nielsen SJ (2003). The sweetening of the world’s diet. Obes Res.

[3] Bray GA, Fructose. Pure, White, and Deadly? Fructose, by Any Other Name, Is a Health Hazard. J Diabetes Sci Technol. 2010;4(4). 10.1177/193229681000400432 PMC2909535 20663467

[4] Malik VS, Hu FB (2022). The role of sugar-sweetened beverages in the global epidemics of obesity and chronic diseases. Nat Reviews Endocrinol.

[5] Malik VS, Pan A, Willett WC, Hu FB (2013). Sugar-sweetened beverages and weight gain in children and adults: a systematic review and meta-analysis. Am J Clin Nutr.

[6] Basu S, McKee M, Galea G, Stuckler D (2013). Relationship of soft drink consumption to global overweight, obesity, and diabetes: a cross-national analysis of 75 countries. Am J Public Health.

[7] Singh GM, Micha R, Khatibzadeh S, Shi P, Lim S, Andrews KG, Engell RE, Ezzati M, Mozaffarian D, Global (2015). Regional, and National Consumption of Sugar-Sweetened Beverages, Fruit Juices, and milk: a systematic Assessment of Beverage Intake in 187 countries. PLoS ONE.

[8] Ervin RB. Consumption of added sugar among US children and adolescents, 2005–2008: US Department of Health & Human Services, Centers for Disease Control and ...; 2012.

[9] Popkin BM (2010). Patterns of beverage use across the lifecycle. Physiol Behav.

[10] Lundeen EA, Park S, Woo Baidal JA, Sharma AJ, Blanck HM (2020). Sugar-Sweetened Beverage Intake among pregnant and non-pregnant women of Reproductive Age. Matern Child Health J.

[11] Yudkin J, Pure. white and deadly:Penguin; 1988.

[12] Bleich SN, Vercammen KA (2018). The negative impact of sugar-sweetened beverages on children’s health: an update of the literature. BMC Obes.

[13] Malik VS, Willett WC, Hu FB (2009). Sugar-sweetened beverages and BMI in children and adolescents: reanalyses of a meta-analysis. Am J Clin Nutr.

[14] Vartanian LR, Schwartz MB, Brownell KD (2007). Effects of soft drink consumption on nutrition and health: a systematic review and meta-analysis. Am J Public Health.

[15] Malik VS, Hu FB (2019). Sugar-Sweetened Beverages and Cardiometabolic Health: an update of the evidence. Nutrients.

[16] Exercise during pregnancy and the postpartum period (2003). Clin Obstet Gynecol.

[17] Malik VS, Schulze MB, Hu FB (2006). Intake of sugar-sweetened beverages and weight gain: a systematic review. Am J Clin Nutr.

[18] Luger M, Lafontan M, Bes-Rastrollo M, Winzer E, Yumuk V, Farpour-Lambert N (2017). Sugar-Sweetened Beverages and Weight Gain in children and adults: a systematic review from 2013 to 2015 and a comparison with previous studies. Obes Facts.

[19] DiNicolantonio JJ, O’Keefe JH, Wilson WL (2018). Sugar addiction: is it real? A narrative review. Br J Sports Med.

[20] Avena NM, Rada P, Hoebel BG (2008). Evidence for sugar addiction: behavioral and neurochemical effects of intermittent, excessive sugar intake. Neurosci Biobehav Rev.

[21] Schulze MB, Liu S, Rimm EB, Manson JE, Willett WC, Hu FB (2004). Glycemic index, glycemic load, and dietary fiber intake and incidence of type 2 diabetes in younger and middle-aged women. Am J Clin Nutr.

[22] AlEssa HB, Ley SH, Rosner B, Malik VS, Willett WC, Campos H, Hu FB (2016). High Fiber and low starch intakes are Associated with circulating intermediate biomarkers of type 2 diabetes among women. J Nutr.

[23] Malik VS, Popkin BM, Bray GA, Després J-P, Willett WC, Hu FB (2010). Sugar-Sweetened Beverages and risk of metabolic syndrome and type 2 diabetes: a meta-analysis. Diabetes Care.

[24] Hu FB, Malik VS (2010). Sugar-sweetened beverages and risk of obesity and type 2 diabetes: epidemiologic evidence. Physiol Behav.

[25] Xi B, Huang Y, Reilly KH, Li S, Zheng R, Barrio-Lopez MT, Martinez-Gonzalez MA, Zhou D (2015). Sugar-sweetened beverages and risk of hypertension and CVD: a dose–response meta-analysis. Br J Nutr.

[26] Malik VS, Popkin BM, Bray GA, Després J-P, Hu FB (2010). Sugar-Sweetened beverages, obesity, type 2 diabetes Mellitus, and Cardiovascular Disease Risk. Circulation.

[27] Nseir W, Nassar F, Assy N (2010). Soft drinks consumption and nonalcoholic fatty liver disease. World J Gastroenterol.

[28] WHO, Guideline. Sugars Intake for Adults and Children. WHO website. 2015. 25905159

[29] Casas R, Castro Barquero S, Estruch R (2020). Impact of sugary food consumption on pregnancy: a review. Nutrients.

[30] Gamba RJ, Leung CW, Petito L, Abrams B, Laraia BA (2019). Sugar sweetened beverage consumption during pregnancy is associated with lower diet quality and greater total energy intake. PLoS ONE.

[31] Sánchez-Pimienta TG, Batis C, Lutter CK, Rivera JA (2016). Sugar-Sweetened Beverages are the Main sources of added Sugar Intake in the Mexican Population. J Nutr.

[32] Chen L, Hu FB, Yeung E, Willett W, Zhang C (2009). Prospective study of pre-gravid sugar-sweetened beverage consumption and the risk of gestational diabetes mellitus. Diabetes Care.

[33] Maslova E, Halldorsson TI, Astrup A, Olsen SF (2015). Dietary protein-to-carbohydrate ratio and added sugar as determinants of excessive gestational weight gain: a prospective cohort study. BMJ open.

[34] Renault KM, Carlsen EM, Nørgaard K, Nilas L, Pryds O, Secher NJ, Olsen SF, Halldorsson TI (2015). Intake of sweets, snacks and soft drinks predicts Weight Gain in obese pregnant women: detailed analysis of the results of a Randomised Controlled Trial. PLoS ONE.

[35] Malik AH, Akram Y, Shetty S, Malik SS, Yanchou Njike V (2014). Impact of Sugar-Sweetened Beverages on blood pressure. Am J Cardiol.

[36] Englund-Ögge L, Brantsæter AL, Haugen M, Sengpiel V, Khatibi A, Myhre R, Myking S, Meltzer HM, Kacerovsky M, Nilsen RM, Jacobsson B (2012). Association between intake of artificially sweetened and sugar-sweetened beverages and preterm delivery: a large prospective cohort study. Am J Clin Nutr.

[37] Halldorsson TI, Strøm M, Petersen SB, Olsen SF (2010). Intake of artificially sweetened soft drinks and risk of preterm delivery: a prospective cohort study in 59,334 danish pregnant women. Am J Clin Nutr.

[38] Hu Z, Tylavsky FA, Kocak M, Fowke JH, Han JC, Davis RL, LeWinn KZ, Bush NR, Sathyanarayana S, Karr CJ, Zhao Q (2020). Effects of maternal dietary patterns during pregnancy on early childhood growth trajectories and obesity risk: the CANDLE study. Nutrients.

[39] Gillman MW, Rifas-Shiman SL, Fernandez-Barres S, Kleinman K, Taveras EM, Oken E. Beverage Intake During Pregnancy and Childhood Adiposity. Pediatrics. 2017;140(2). 10.1542/peds.2017-0031 PMC5527670 28689188

[40] Perrar I, Schmitting S, Della Corte KW, Buyken AE, Alexy U (2020). Age and time trends in sugar intake among children and adolescents: results from the DONALD study. Eur J Nutr.

[41] Jen V, Erler NS, Tielemans MJ, Braun KV, Jaddoe VW, Franco OH, Voortman T (2017). Mothers’ intake of sugar-containing beverages during pregnancy and body composition of their children during childhood: the Generation R Study. Am J Clin Nutr.

[42] Bédard A, Northstone K, Henderson AJ, Shaheen SO (2017). Maternal intake of sugar during pregnancy and childhood respiratory and atopic outcomes. Eur Respir J.

[43] Cohen JFW, Rifas-Shiman SL, Young J, Oken E (2018). Associations of prenatal and child Sugar Intake with Child Cognition. Am J Prev Med.

[44] Chang M-W, Lin CJ, Lee RE, Wegener DT, Hu J, Williams KP (2022). Factors Associated with Beverage Intake in Low-Income, overweight, or obese pregnant women. Nutrients.

[45] Oyewole OE, Atinmo T. Nutrition transition and chronic diseases in Nigeria. The Proceedings of the Nutrition Society. 2015;74(4):460-5. 10.1017/S0029665115002402 26242780

[46] Bosu WK (2015). An overview of the nutrition transition in West Africa: implications for noncommunicable diseases. Proc Nutr Soc.

[47] Mustapha AM, Fakokunde TO, Awolusi OD (2014). The quick service restaurant business in Nigeria: exploring the emerging opportunity for entrepreneurial development and growth. Global J Commer Manage Perspective.

[48] Adeoye IA, Bamgboye EA, Omigbodun AO (2022). The Ibadan pregnancy cohort study (IbPCS), a prospective cohort study protocol. Afr J biomedical Res.

[49] Adeoye IA. Effect of Maternal Obesity, lifestyle characteristics on glycaemic control, gestational weight gain and pregnancy outcomes in Ibadan, Nigeria. PhD Dissertation, University of Ibadan, Nigeria. 2021.

[50] Chasan-Taber L, Schmidt MD, Roberts DE, Hosmer D, Markenson G, Freedson PS (2004). Development and validation of a pregnancy physical activity questionnaire. Med Sci Sports Exerc.

[51] WHO. Overweight and Obesity. World health Organization. 2018.

[52] WHO. Overweight and obesity. World Health Organization; 2020.

[53] Cohen S, Kamarck T, Mermelstein R (1983). A global measure of perceived stress. J Health Soc Behav.

[54] Calcaterra V, Cena H, Magenes VC, Vincenti A, Comola G, Beretta A, Di Napoli I, Zuccotti G (2023). Sugar-Sweetened Beverages and metabolic risk in children and adolescents with obesity: a narrative review. Nutrients.

[55] Sigala DM, Stanhope KL (2021). An exploration of the role of Sugar-Sweetened Beverage in promoting obesity and health disparities. Curr Obes Rep.

[56] Cheng ER, Batista E, Chen L, Nichols K, Park S, Charles N, Woo Baidal J (2020). Correlates of sugar-sweetened beverage intake among low-income women during the first 1000 days. Public Health Nutr.

[57] Wright KM, Dono J, Brownbill AL, Pearson O, Bowden J, Wycherley TP, Keech W, O’Dea K, Roder D, Avery JC, Miller CL (2019). Sugar-sweetened beverage (SSB) consumption, correlates and interventions among australian Aboriginal and Torres Strait Islander communities: a scoping review. BMJ open.

[58] Thow A-M, Erzse A, Asiki G, Ruhara CM, Ahaibwe G, Ngoma T, Amukugo HJ, Wanjohi MN, Mukanu MM, Gaogane L, Abdool Karim S, Hofman K (2021). Study design: policy landscape analysis for sugar-sweetened beverage taxation in seven sub-saharan african countries. Global Health Action.

[59] Bowman SA, Gortmaker SL, Ebbeling CB, Pereira MA, Ludwig DS (2004). Effects of fast-food consumption on energy intake and diet quality among children in a national household survey. Pediatrics.

[60] Wrottesley SV, Pisa PT, Norris SA. The Influence of Maternal Dietary Patterns on Body Mass Index and Gestational Weight Gain in Urban Black South African Women. Nutrients. 2017;9(7). 10.3390/nu9070732 PMC5537846 28696364

[61] Hu FB (2013). Resolved: there is sufficient scientific evidence that decreasing sugar-sweetened beverage consumption will reduce the prevalence of obesity and obesity-related diseases. Obes reviews: official J Int Association Study Obes.

[62] IOM. Weight Gain During Pregnancy. : Reexamining the Guidelines In: Rasmussen KM, Yaktine AL, editors. The National Academies Collection: Reports funded by National Institutes of Health. Washington (DC): National Academies Press (US) Copyright © 2009, National Academy of Sciences.; 2009. 20669500

[63] de Koning L, Malik VS, Kellogg MD, Rimm EB, Willett WC, Hu FB (2012). Sweetened beverage consumption, incident coronary heart disease, and biomarkers of risk in men. Circulation.

[64] Park S, Pan L, Sherry B, Blanck HM (2014). Consumption of sugar-sweetened beverages among US adults in 6 states. Behav Risk Factor Surveillance Syst 2011 Preventing chronic disease.

[65] Kristal RB, Blank AE, J W-R PAS. Factors Associated With Daily Consumption of Sugar-Sweetened Beverages Among Adult Patients at Four Federally Qualified Health Centers, Bronx, New York, 2013. CDC Prev Chronic Dis. 2015;12(140342). 10.5888/pcd12.140342 PMC4290096 25569695

[66] Guthrie JF, Lin BH, Frazao E (2002). Role of food prepared away from home in the American diet, 1977-78 versus 1994-96: changes and consequences. J Nutr Educ Behav.

[67] Skreden M, Bere E, Sagedal LR, Vistad I, Øverby NC (2015). Changes in beverage consumption from pre-pregnancy to early pregnancy in the norwegian fit for delivery study. Public Health Nutr.

[68] Alcaraz A, Pichon-Riviere A, Palacios A, Bardach A, Balan DJ, Perelli L, Augustovski F, Ciapponi A (2021). Sugar sweetened beverages attributable disease burden and the potential impact of policy interventions: a systematic review of epidemiological and decision models. BMC Public Health.

[69] Krieger J, Bleich SN, Scarmo S, Ng SW (2021). Sugar-Sweetened Beverage reduction policies: Progress and Promise. Annu Rev Public Health.

[70] Stookey JD, Constant F, Gardner CD, Popkin BM (2007). Replacing sweetened caloric beverages with drinking water is associated with lower energy intake. Obes (Silver Spring Md).

[71] Daniels MC, Popkin BM (2010). Impact of water intake on energy intake and weight status: a systematic review. Nutr Rev.

[72] Bank W. Taxes on Sugar-Sweetened Beverages: International evidence and experiences. World Bank; 2020.

[73] WHO. Fiscal policies for diet and the prevention of noncommunicable diseases. WHO. 2015.

[74] Thow AM, Abdool Karim S, Mukanu MM, Ahaibwe G, Wanjohi M, Gaogane L, Amukugo HJ, Ruhara CM, Ngoma T, Asiki G, Erzse A, Hofman K (2021). The political economy of sugar-sweetened beverage taxation: an analysis from seven countries in sub-saharan Africa. Global Health Action.

[75] Ndlovu N, Swinburn B (2021). Readiness for sugar sweetened beverage taxation in sub-saharan Africa. Global Health Action.

[76] Audain K, Levy L, Ellahi B. Sugar-sweetened beverage consumption in the early years and implications for type-2 diabetes: a sub-Saharan Africa context. Proceedings of the Nutrition Society. 2019;78(4):547 – 53. 10.1017/S0029665118002860 30816084

