# Supplementary material for: Sugar-sweetened beverage consumption among pregnant women attending general and teaching hospitals in Ibadan, Nigeria: SSB consumption during pregnancy
Source: BMC Public Health. 2023 May 26;23:980. doi: 10.1186/s12889-023-15828-z (PMC10224601; doi:10.1186/s12889-023-15828-z)
Supplement: Supplementary file 1 — Supplementary Material 1 [file 12889_2023_15828_MOESM1_ESM.docx]

**Figure 3: Distribution of Sugar sweetened beverages consumption among pregnant women in Ibadan.- Remove – put in supplementary**
